# MALE AND FEMALE WORKERS SUFFERING FROM CHRONIC LOW BACK PAIN DISPLAY DIFFERENT INTERRELATIONSHIPS BETWEEN THE BIOPSYCHOSOCIAL VARIABLES

**DOI:** 10.2340/jrm.v57.43450

**Published:** 2025-09-04

**Authors:** Charlotte LANHERS, Christian DUALÉ, Alice CORTEVAL, Emmanuel COUDEYRE, Bruno PEREIRA, Nicolas KERCKHOVE

**Affiliations:** 1 CHU Clermont-Ferrand, Médecine Physique et Réadaptation, Clermont-Ferrand; 2 Université Clermont-Auvergne, Clermont-Ferrand; 3 CHU Clermont-Ferrand, Plateforme d’Investigation Clinique (INSERM CIC1405), Clermont-Ferrand; 4 INSERM UMR1107 Neuro-Dol, Clermont-Ferrand; 5 Institut Analgesia, Clermont-Ferrandce; 6 INRAE UMR1019 “Nutrition Humaine”, Saint-Genès-Champanelle; 7 CHU Clermont-Ferrand, Direction de la Recherche Clinique et des Innovations, Clermont-Ferrand; 8 CHU Clermont-Ferrand, Pharmacologie Médicale, Clermont-Ferrand, France

**Keywords:** biopsychosocial, gender, low back pain, sex, work, cross-sectional survey

## Abstract

**Objective:**

To study the biopsychosocial model of chronic low back pain in the workplace and the role of sex in it.

**Design:**

Cross-sectional nationwide survey in a service company.

**Patients:**

256 workers (women 64.1%) reporting chronic low back pain.

**Methods:**

Variables on biometry, job description, physical activity, pain severity/interference, neuropathic features, and questionnaire-based cognitive and affective parameters were collected. Within each sex group, the interrelationships between variables by Multiple Correspondence Analysis were analysed, followed by cluster analysis.

**Results:**

In the overall sample, neuropathic features were reported by 28.9% of the patients; the cluster including the high pain disorder modalities (i.e., severity and interference) also included high pain catastrophizing and fear/avoidance towards work, as well as neuropathic features. However, in men, the modalities neighbouring high pain disorder were high anxiety and depression, and low mental quality of life, while in women, they were kinesiophobia, high fear/avoidance towards physical activity and stress at work, and low physical quality of life.

**Conclusion:**

As there is now a major demand for defining chronic low back pain patients based on their biopsychosocial profile to improve care and prognosis, this study’s results indicate the relevance of conducting such phenotyping at an early stage in a working environment, and that it is preferable to construct predictive models for each sex group.

## LAY ABSTRACT

We carried out a survey of workers in a nationwide service company, and collected information from those who claimed to suffer from chronic low-back pain, i.e., for more than 3 months. Factor analyses showing the interrelationships between different medical, psychological, and social aspects of these people not only confirmed the strong interrelationship between these different markers, but also identified different behaviours depending on the sex of the people suffering from low back pain. Indeed, whereas in men the severity of low-back pain was more closely linked to signs of general psychological problems, in women the related problems were of a more physical nature.

Chronic low back pain (cLBP) is the most frequent form of chronic pain and a major contributor to disability worldwide (1, 2). While mechanical factors and musculoskeletal lesions have an undisputed role in the genesis of low back pain (LBP), its trend towards chronicity should be considered within the broader framework of biophysical, psychological, and social factors, which all interact. In this biopsychosocial model, both affective (e.g., anxiety, depression, or stress) and cognitive factors are involved (3–5). Among the cognitive factors, pain catastrophizing (difficulty in coping with painful situations) is associated with pain intensity, disability, and treatment failure in cLBP (6, 7), while kinesiophobia (fear of movement due to the expectation of pain or re-injury) can lead the patient to avoid certain activities and aggravate disability (8). Furthermore, either sex *per se* (i.e., biological differentiation) or gender (i.e., psychosocial differentiation) may interact with all the other biopsychosocial aspects of pain (9, 10), and there is a high need for a better understanding of the role of sex differences in the mechanisms of chronic pain across the lifespan (11). A systematic approach to the effect of sex and gender in biomedical research is nowadays strongly encouraged (12).

In addition, while the consequences of cLBP on work activity have been studied extensively, less is known about the true role of work as a primary cause or aggravating factor (13). A survey conducted 23 years ago on French workers reported that cLBP was affected by physical constraints, with a low influence of sex (14). However, the interacting roles of psychosocial factors was unknown at that time, and the types of jobs carried out in Western countries have changed considerably since then. Recently, we conducted a cross-sectional survey of the prevalence and features of chronic pain disorders in IKEA France workers; about one-quarter of the surveyed sample reported cLBP (15). As several biometric, job-related, affective and cognitive parameters were recorded in this survey, it offered us the possibility of studying the interrelationships within those variables. Moreover, a well-balanced sex ratio in our sample allowed us to study men and women separately, in order to identify whether the interrelationships were the same in both groups. By doing so, we wished to conduct a by-sex holistic observation of cLBP cases before ongoing psychosocial processes (such as depression, catastrophism, or long-term sick leave) had exerted too much influence on the painful disorder, as in the case of patients undergoing specialized care. Our primary objective was therefore to study the interrelationships within the biopsychosocial model in workers suffering from cLBP, and particularly whether these interrelationships were different in men and women.

## METHODS

This is a secondary analysis conducted on data obtained from a cross-sectional survey with wider epidemiological purposes, whose methods and results have already been published (15). The survey targeted all the adult employees of 14 IKEA France stores, representative of the workforce of IKEA France. The way the survey was designed and conducted is detailed in Appendix S1 (part A).

The first part of the survey was a core set of personal questions, which all participants were asked to answer: sex, age, weight, height, level of declared physical activity, and job description (see Appendix S1, part B). As the question to define sex was simply “are you: a man?/a woman?”, and neither asked how the participant identified him-/herself nor offered other options such as “non-binary” or “other”, we considered that the resulting variable was representative of sex rather than gender. This was followed by psychometric questionnaires, such as the Hospital Anxiety and Depression Scale (HADS) (16) and the Pain Catastrophizing Scale (PCS) (17), followed by an assessment of stress at home and at work by an electronic visual analogue scale (18, 19). The participant then had to answer the question, “Do you suffer from pain at the moment?”. If the answer was “yes”, then the participant was asked to state whether the pain had lasted for more than 3 months, and to complete a set of questionnaires to describe this pain as well as other fields related to it: severity and interference (impact) of pain with the Brief Pain Inventory (BPI) (20), neuropathic pain screening with the DN4 questionnaire (21, 22); health-related quality-of-life (QoL) with the SF*-*12 (23); fears and beliefs concerning pain with the Tampa Scale of Kinesiophobia (TSK) (24); the Fear Avoidance Beliefs Questionnaire (FABQ), which assesses both fear and avoidance towards physical activity (FAPA) and work (FAW) (25). We did not use any cLBP-specific questionnaire, but the BPI pain interference score is a good surrogate for cLBP-induced disability (26). The structure, interpretation, and references for the validated French version of all questionnaires are detailed in Appendix S1. The participants were also asked to self-define the cause(s) of chronic pain, and whether he/she perceived work as worsening it; he/she could also provide additional details for these last 2 items, as well as concerning the type of pain, by means of a free-text field. Data were extracted as Excel files (Microsoft Office Excel 2010; Microsoft Corp, Redmond, WA, USA). A pain physician (CD) reviewed the free responses regarding the type of pain – as well as those concerning the BPI and the DN4 – and interpreted them for each participant. From this, the physician could define (*i*) the case as LBP if low back was clearly identified as the only or principal source of pain; (*ii*) the features of pain (neuropathic if positive DN4, mixed if typical neuropathic symptoms were noted despite a negative DN4, nociplastic if associated with widespread pain, nociceptive otherwise, and undefined if information from DN4 was incomplete); and (*iii*) whether work was perceived as a cause of the pain or as worsening it. A binary variable called “neuropathic features” was defined as either neuropathic or mixed cLBP. The body mass index (BMI) was calculated from the height and weight. Finally, only those cases reporting an average cLBP severity (from the BPI) > 30/100 and for at least 3 months (i.e., moderate-to-severe) were extracted for analysis.

Data analysis and graph construction was performed using Microsoft Office Excel 2010, XLStat (Addinsoft, Paris, F) and Microsoft Office PowerPoint 2010 (Microsoft Corp, Redmond, WA, USA). For descriptive purposes, numerical data were expressed as mean± SD for the Gaussian distribution or as quartiles otherwise, and nominal data were expressed as counts and percentages. Patients or variables with an incomplete dataset were discarded. To compare the numerical variables between the 2 sex groups, we used either Student’s *t*-test or the Mann–Whitney test depending on distribution (checked by a Q–Q plot); for nominal variables, we used either the χ^2^ test or Fisher’s exact test depending on distribution.

We first conducted correlation analyses in which each variable was tested against each of the others, this within the whole sample and within each sex group (see Appendix S1, part C). This helped to view the sample’s behaviour according to the biopsychosocial model of cLBP, and to spot the collinearities (i.e., very strong correlations between 2 variables) to discard the redundant variables. The main analyses we conducted to study the interrelationships between all the biopsychosocial variables were factorial multivariable analyses within each sex group. Rather than to identify the putative cause of a given outcome, their aim was to summarize the interrelationships within 1 single dimension. Since this was a transversal survey focused on chronic pain, we did not have a strong hypothesis for causalities between variables. Furthermore, as many variables were of a psychosocial nature, multiple correlations were expected, and multicollinearity could have impaired the statistical power of inferential analyses. We therefore opted for Multiple Correspondence Analysis (MCA), in which the numerical variables were transformed into nominal ordinal ones. This categorization is a way to reinforce associations, especially if the relationship is not linear; relevance is not affected as long as categorization is based on logical processes. Categorization can even improve relevance because it focuses on the interrelationships between the classes (modalities) representing the states of highest disorder or risk (i.e., the highest pain interference, the highest kinesiophobia, and so on), states which are known to predict the severity of cLBP (i.e., poor response to treatment or ongoing sick leave) (27, 28). The method of categorization is detailed in **[Table T0001]T**able I. The MCAs included: (*i*) selection of the best representative factors to obtain a total percentage of acceptable variance, (*ii*) calculation of the contribution of each category to each factor, (*iii*) construction of a 2-dimensional graph plotting the principal coordinates of each variable/modality on each main axis (“factor”), and (*iv*) analysis of the proximity between variables/modalities. As our primary objective was to study the effect of sex, we conducted MCAs within each sex group; as the distribution of variables could vary with sex, the classification was made for each sex-specific group analysis (Table I). For each MCA, we studied the proximity between modalities by cluster analysis of the principal coordinates on the main factorial axes, with a focus on the modalities representing the states of highest disorder/risk as mentioned above, and especially the modality of highest pain interference (3rd tercile), as a cLBP-induced disability is a known predictor of poor response to treatment or ongoing sick leave (27, 28). Three clustering methods were used in parallel: ascending hierarchical classification, k-means, and univariate clustering (see Appendix S1, part D). The final cluster representing the worst state of cLBP included those modalities which belonged to the cluster including the highest pain interference in the 3 methods. The sex differences were assessed by a simple observation of the final cluster for each sex.

**Table I T0001:** Categorization into classes of the numerical variables

Original variable	Type	Women	Men
Age (years)	TER	< 35/[35–45]/> 45	< 36/[36–45]/> 45
BMI (kg/m^–2^)	REF	< 25 (normal)/[25–30[ (overweight)/≥ 30 (obesity)	
Declared physical activity	MM	none or 1 h/week (low)/2–3 h/week (moderate)/≥ 4 h/week (high)	
Seniority within the job (months)	MM	< 70/[70–170]/> 170	< 69/[69–160]/> 160
Time spent at work	MM	< 30 h/week (partial)/≥ 30 h/week (full)	
Anxiety (HADS, out of 21)	REF	< 8/[8–11]/> 11	
Depression (HADS, out of 21)	REF	< 8/[8–11]/> 11	
Pain catastrophizing (PCS, out of 52)	TER	< 12/[12–24]/> 24	< 13/[13–24]/> 24
Stress at home (scale, out of 100)	TER	< 22/[22–40]/> 40	< 20/[20–37]/> 37
Stress at work (scale, out of 100)	TER	< 51/[51–70[/≥ 70	< 61/[61–74[/≥ 74
Pain severity (BPI, out of 10)	TER	< 3.8/[3.8–5.6]/> 5.6	< 4.1/[4.1–5.4]/> 5.4
Pain interference (BPI, out of 10)	TER	< 2.6/[2.6–5.2]/> 5.2	< 2.5/[2.5–5.3]/> 5.3
Physical quality of life (SF-12, out of 100)	TER	< 40/[40–49.1]/> 49.1	< 41/[41–48.3]/> 48.3
Mental quality of life (SF-12, out of 100)	TER	< 38/[38–48]/> 48	< 37.5/[37.5–47]/> 47
Kinesiophobia (TSK, out of 68)	REF	< 40 (negative)/≥ 40 (positive)	
FAPA (FABQ, out of 24)	TER	< 8/[8–14]/> 14	< 11/[11–15]/> 15
FAW (FABQ, out of 42)	TER	< 10/[10–23]/> 23	< 18/[18–27]/> 27

Description of the way the numerical variables were categorized into classes for purposes of factorial analyses. For categorization of scores, we used a validated method when available and relevant for the clinical condition; for the BMI, the definitions of overweight and obesity are those of the World Health Organization. When no method was referenced, the distribution of the variables within the main sample was plotted onto scattergrams; if several modalities appeared, cut-off values were defined to separate them (either at the point of lowest density, or at the midway point between peaks); otherwise, the sample was separated into terciles. When categorizations were trimodal, the classes were named either as 1st/2nd/3rd tercile, or as ordinal values (e.g., low/intermediate/high); otherwise, the names of the classes are given in the table. BMI: body mass index; BPI: Brief Pain Inventory; FABQ: Fear Avoidance Back Questionnaire; FAPA: fear/avoidance towards physical activity; FAW: fear/avoidance towards work; HADS: Hospital Anxiety and Depression Scale; MM: according to a multimodal distribution; PCS: Pain Catastrophizing Scale; REF: referenced classification; TER: classification into terciles; TSK: Tampa Scale of Kinesiophobia.

Finally, as such results had been reported in the main study (15) or in the previous ESTEV study conducted in French workers (14), we tested the relationship with pain interference of several variables, namely the fact of not having consecutive rest days, the types of repetitive gestures, the history of sick leave, and the perception of work as causing or worsening cLBP. We restricted the analysis to those gestures likely to favour cLBP, namely back torsion/flexion, exposure to vibrations, moving heavy loads, static standing and sitting position, and extended work on screens (3, 14, 29–31). Between-groups comparisons were conducted by a Mann–Whitney test.

As this was a secondary analysis, the sample size had not been predefined, but we estimated it to be sufficient to carry out multivariable factorial analyses, as long as the number of variables did not exceed one-tenth of the sample size.

## RESULTS

A total of 256 cases met the selection criteria and could be used for analysis; they are described in Table II. Several observations can be made about the whole sample. Repetitive gestures at work were reported in most of the cases (89.5%), with each type identified in about half of the cases, except exposure to vibrations and static sitting position, which were present in about one-quarter. Signs of anxious and depressive symptoms were noted in about one-half and one-quarter of the cases, respectively. Both physical and mental QoL were average, with no more than one-quarter of responders reporting component summaries over 50/100. Pain interference was slightly lower than pain severity, and half of the cases reported relevant kinesiophobia. Neuropathic features were identified in 28.9% of cases. The relationship with work also appeared to be disturbed, with high median stress at work (65/100), and about 80% of responders perceiving work as a cause of their cLBP, and as worsening it. In this sample of cLBP, the ratio of women (164/259, 64.1%) was higher than in the survey responders who declared having no pain (262/511, 51.3%; *p* = 0.001, χ^2^ test). Significant differences between the sex groups were also noted for several variables. Men were more overweight than women, and had higher levels of depression, stress at work, kinesiophobia, and FAW. The declaration of repetitive gesture was equal for both sexes, but more repetitive gestures were reported by men, with the biggest gap being for exposure to vibrations. Moving heavy loads and static standing position were reported equally by men and women.

**Table II:** Description of the study sample

Factor	Whole	Women	Men	*p*-value
Sample size	256	164	92	
Demography and morphometry				
Age (years)	41 (32–47)^a^	41 (32–48)	40 (32–46)	0.694
Weight (kg)	72 (61–85)	65 (59–76)	83 (75–90)	NC
Height (cm)	170 (165–176)	166 (160–170)	179 (173–185)	NC
BMI (kg/m^–2^)	24.6 (22–27.7)	23.4 (21.3–27.3)	25.5 (23.5–28.6)	0.003
BMI > 25 k/m^–2^ (i.e., overweight or obesity)	117 (45.7)	64 (39.0)	53 (57.6)	0.004
BMI > 30 kg/m^–2^ (i.e., obesity)	42 (16.4)	25 (15.2)	17 (18.5)	0.503
Declared physical activity (h per week)				0.129
None	125 (48.8)	88 (53.7)	37 (40.2)	
≤1	30 (11.7)	17 (10.4)	13 (14.1)
2–3	67 (26.2)	41 (25.0)	26 (28.3)
4–5	17 (6.6)	11 (6.7)	6 (6.5)
> 5	17 (6.6)	7 (4.3)	10 (10.9)
Job description				
Seniority (months)				
Within the company	120 (36–168)	120 (36–168)	120 (44–156)	0.827
Within the job	84 (30–144)	84 (32–134)	86 (28–144)	0.975
Job description				1.000
Sales	75 (29.3)	44 (26.8)	31 (33.7)	
Logistics	48 (18.8)	22 (13.4)	26 (28.3)
Catering	27 (10.5)	19 (11.6)	8 (8.7)
Fittings	27 (10.5)	23 (14.0)	4 (4.3)
Administration	25 (9.8)	17 (10.4)	8 (8.7)
Recovery	7 (2.7)	2 (1.2)	5 (5.4)
Maintenance	4 (1.6)	0 (0.0)	4 (4.3)
Miscellaneous ^b^	43 (16.8)	37 (22.6)	6 (6.5)
Repetitive gestures (declared)				
Arms/shoulders raised	165 (64.5)	95 (57.9)	70 (76.1)	0.004
Wrist torsion	134 (52.3)	78 (47.6)	56 (60.9)	0.041
Wrist flexion	135 (52.7)	75 (45.7)	60 (65.2)	0.003
Neck torsion	133 (52.0)	80 (48.8)	53 (57.6)	0.176
Neck flexion	119 (46.5)	67 (40.9)	52 (56.5)	0.016
Back torsion	174 (68.0)	103 (62.8)	71 (77.2)	0.018
Back flexion	173 (67.6)	101 (61.6)	72 (78.3)	0.006
Exposure to vibrations	57 (22.3)	20 (12.2)	37 (40.2)	< 0.0001
Moving heavy loads	169 (66.0)	102 (62.2)	67 (72.8)	0.085
Static standing position	122 (47.7)	81 (49.4)	41 (44.6)	0.460
Static sitting position	69 (27.0)	37 (22.6)	32 (34.8)	0.035
Extended work on screen	126 (49.2)	82 (50.0)	44 (47.8)	0.740
Any repetitive gesture	229 (89.5)	143 (87.2)	86 (93.5)	0.117
Time spent at work (h/week)	35 (35–35)	35 (33–35)	35 (35–35)	0.152
Time spent on travel to work (min/day)	20 (15–30)	20 (15–30)	23 (15–30)	0.553
Consecutive days of rest	113 (44.1)	73 (44.5)	40 (43.5)	0.873
History of sick leave	154 (60.2)	98 (59.8)	56 (60.9)	0.861
Health-related quality-of-life (SF-12)				
Physical component summary (score out of 100)	45.1 (37.8–50.8)	46 (37–52)	44 (39–50)	0.591
Mental component summary (score out of 100)	42.1 (35–50)	43 (35–50)	42 (34–49)	0.614
Severity of low back pain (BPI)				
Worst pain in last 24 h (scale 0–100)	65 (50–80)	65 (47–80)	65 (55–77)	NC
Least pain in last 24 h (scale 0–100)	24 (10–40)	23 (10–40)	25 (10–37)	
Pain on average (scale 0–100)	55 (41–65)	54 (40–64)	56 (41–66)	
Pain right now (scale 0–100)	47 (25–61)	47 (25–61)	47 (29–60)	
Pain severity score (score 0–10)	4.7 (3.4–5.9)	5 (3–6)	5 (4–6)	0.555
Interference of low back pain (BPI)				
With general activity (scale 0–100)	50 (21–70)	50 (20–67)	51 (21–70)	NC
With mood (scale 0–100)	50 (15–66)	48 (15–63)	51 (23–70)	
With walking ability (scale 0–100)	30 (5–57)	30 (2–57)	40 (10–59)	
With normal work (including housework) (scale 0–100)	50 (20–70)	50 (20–66)	50 (23–70)	
With relations with other people (scale 0–100)	20 (0–51)	20 (0–50)	27 (5–60)	
With sleep (scale 0–100)	52 (15–75)	50 (20–78)	58 (14–75)	
With enjoyment of life (scale 0–100)	15 (0–50)	12 (0–50)	18 (0–51)	
Pain interference score (score 0–10)	4.0 (1.9–5.7)	4.0 (2–6)	4 (2–6)	0.344
Descriptive features of low back pain				
Mainly nociceptive	150 (58.6)	96 (58.5)	54 (58.7)	0.865
Mainly neuropathic	10 (3.9)	5 (3.0)	5 (5.4)	
Mixed nociceptive/neuropathic	64 (25.0)	43 (26.2)	21 (22.8)	
Nociplastic	13 (5.1)	11 (6.7)	2 (2.2)	
Undefined	19 (7.4)	9 (5.5)	10 (10.9)	
Psychometry: affective outcomes				
Anxiety (HADS, out of 21)	8 (5–11)	8 (6–11)	7 (5–11)	0.200
Depression (HADS, out of 21)	5 (2–8)	5 (2–8)	6 (4–9)	0.011
Stress at home (scale 0–100)	30 (15–50)	30 (15–50)	30 (12–41)	0.392
Stress at work (scale 0–100)	65 (50–76)	60 (49–75)	70 (50–80)	0.039
Psychometry: cognitive outcomes				
Pain catastrophizing (PCS, out of 52)	19 (10–30)	18 (9–28)	20 (11–30)	0.320
Kinesiophobia (TSK, out of 68)	40 (33–47)	38 (31–45)	44 (38–48)	< 0.0001
Fear/avoidance towards physical activity (FABQ, out of 24)	12 (7–16)	12 (6–16)	13 (9–17)	0.070
Fear/avoidance towards work (FABQ, out of 42)	19 (10–28)	16 (7–26)	23 (16–30)	0.0004
Work perceived as cause of low back pain	200 (78.1)	125 (76.2)	75 (81.5)	0.325
Work perceived as worsening low back pain	211 (82.4)	134 (81.7)	77 (83.7)	0.688

The numerical data are expressed as medians (1st quartile–3rd quartile), and the nominal values as counts (percentages). BMI: body mass index; BPI: Brief Pain Inventory; FABQ: Fear Avoidance Back Questionnaire; HADS: Hospital Anxiety and Depression Scale; NC: not calculated; PCS: Pain Catastrophizing Scale; SF-12: short form, 12 items; TSK: Tampa Scale of Kinesiophobia.

a Range 20–60 years;

b includes cash register, home delivery, caddy gathering, and various other services.

Among the most noticeable observations for this sample of people suffering from cLBP was that of the 117 cases whose BMI exceeded 25 kg/m^–2^ (i.e., overweight or obesity), 66 (56.4%) stated that they did not do any physical activity. This rate was higher in women (44/64, 68.8%) than in men (22/53, 41.5%) (*p* = 0.003, χ^2^ test).

The Spearman’s correlation matrix (univariate analyses) is shown in Table SI, both for the whole sample group and for each sex group separately. Both in the whole sample and in each sex group, there was a trend for multiple correlations between health-related (e.g., pain or QoL outcomes), affective (e.g., anxiety, depression, or stress) and cognitive (e.g., catastrophizing, fear/avoidance, kinesiophobia) variables. Conversely, the other demographics (e.g., age or BMI) and lifestyle-related factors (e.g., low physical activity or time spent at work) were less frequently correlated not only with health-related, affective, and cognitive variables, but also with each other. Finally, some correlations were observed only in one sex group; for example, stress at home and at work were correlated with age and seniority in men only, while BMI was correlated with pain and QoL outcomes in women only.

**Fig. 1:**
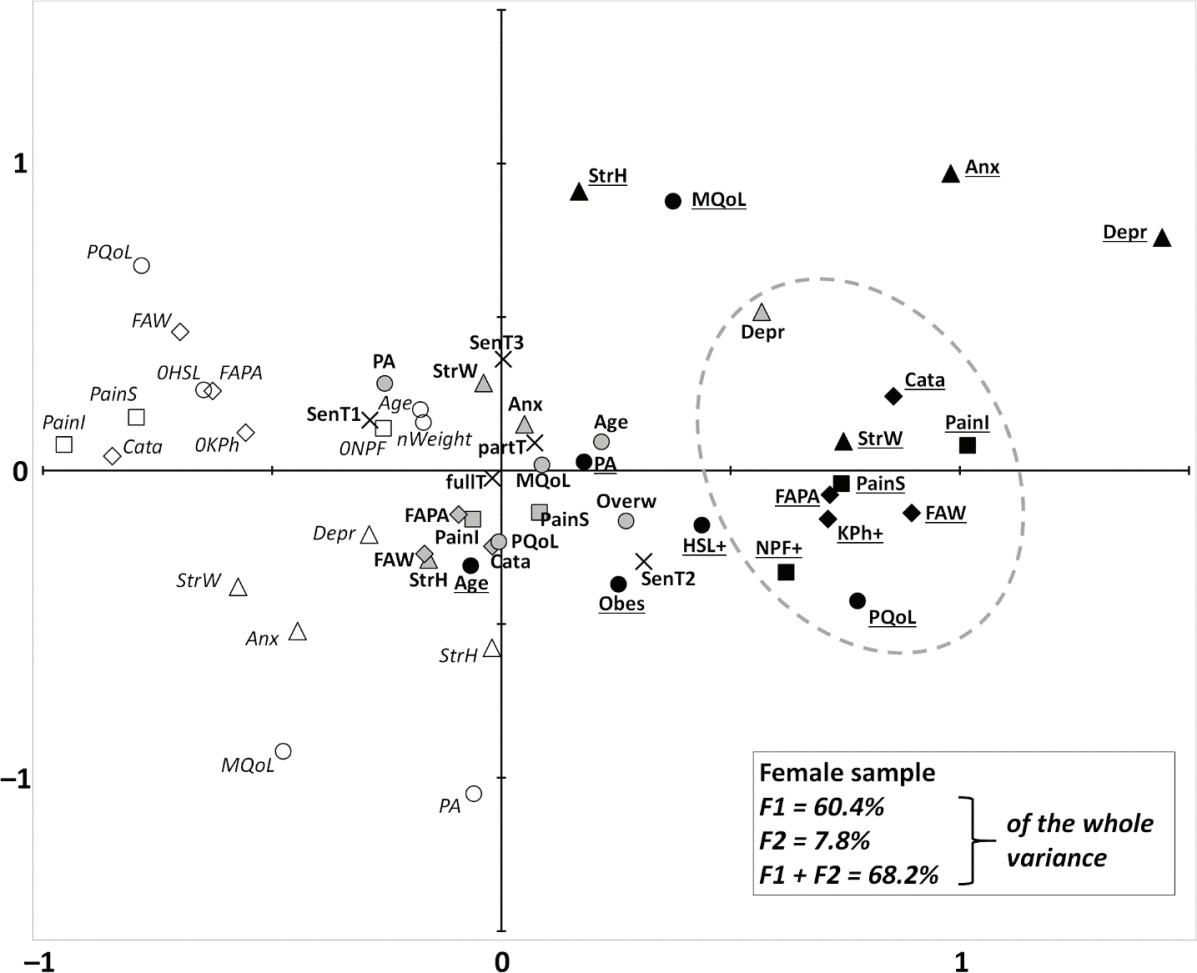
Multiple correspondence analysis of all the female cases who reported chronic low back pain (*n* = 164). This diagram shows the association between the modalities of all the variables of interest. Explanation of how the primarily numerical variables were categorized is given in Table I. Associations are represented in a plane formed by the factorial axes (F1 and F2), representing the majority of the variance. The closer the marks, the more highly associated they are. The modalities of variables directly related to cLBP (represented by squares) are: pain severity (“PainS”) and pain interference (“PainI”), both from the Brief Pain Inventory; and neuropathic features (“NPF”). The modalities of affective variables (represented by triangles) are: anxiety (“Anx”) and depression (“Depr”) from the Hospital Anxiety and Depression scale; and stress at home (“StressH”) and at work (“StressW”), both from analogue visual scales. The modalities of cognitive variables (represented by diamonds) are: pain catastrophizing (“Cata”) from the Pain Catastrophizing Scale; fear/avoidance towards physical activity (“FAPA”) and towards work (“FAW”) from the Fear Avoidance Back Questionnaire; and kinesiophobia from the Tampa Scale of Kinesiophobia. The modalities of the other health-related variables (represented by circles), are: age; declared physical activity (“PA”); body mass index (BMI); and physical and mental quality of life (respectively “PQoL” and “MQoL”), which are the component summaries from the SF-12. As most of the health-related (cLBP or other), affective and cognitive variables are categorized into 3 classes, each corresponding to a level of disorder/risk (low, intermediate, or high), the modalities representing the highest disorder/risk (e.g., 3rd tercile of pain severity or age, 1st tercile of quality of life or physical activity, etc.) are shown as black symbols and bold and underscored font; those representing the lowest level of disorder/risk are shown as white symbols and italic font; those representing the intermediate level of disorder/risk are shown as grey symbols and bold font. The 3 classes of BMI are normal weight (“nWeigh”), overweight (“Overw”) and obesity (“Obes”), with the same respective grade of risk. As neuropathic features and kinesiophobia were classified as presence (“+”) or absence (“0”), they had no intermediate modality. The modalities of the other work-related variables could not be classified by order of disorder/risk; they are represented by crosses, the variables being seniority within the job (“Sen”, in terciles T1–2–3), and weekly time spent at work (“fullT” and “partT” respectively for full and part time). The grey dashed line encloses the final cluster (see Methods and Appendix S1 for details), which includes the 3rd tercile of pain interference and the modalities closely associated with it.

The MCA conducted on the female sample showed that 60.4% of the whole variance was explained by the 1st factorial axis (F1), while the 2nd factorial axis (F2) explained only 7.8% of this, and the subsequent factorial axes made a negligible contribution (4.1% for the 3rd axis). We therefore worked only on the coordinates for factorial axes F1 and F2, representing 68.2% of the whole variance (Fig. 1). On the F1 axis, the indicators of health were well separated (from the states of lowest to highest disorder), while the F2 axis tended to separate affective parameters. However, some parameters – such as age or time spent at work – had a central position on both axes (i.e., they contributed minimally to the overall variance). The results of the cluster analysis are given in Table SII, and illustrated in Fig. 1. The final cluster analysis showed that high pain interference neighboured (by order of proximity): high pain catastrophizing, high FAW, high stress at work, high pain severity, high FAPA, kinesiophobia, low physical QoL, neuropathic features, and intermediate depression.

**Fig. 2:**
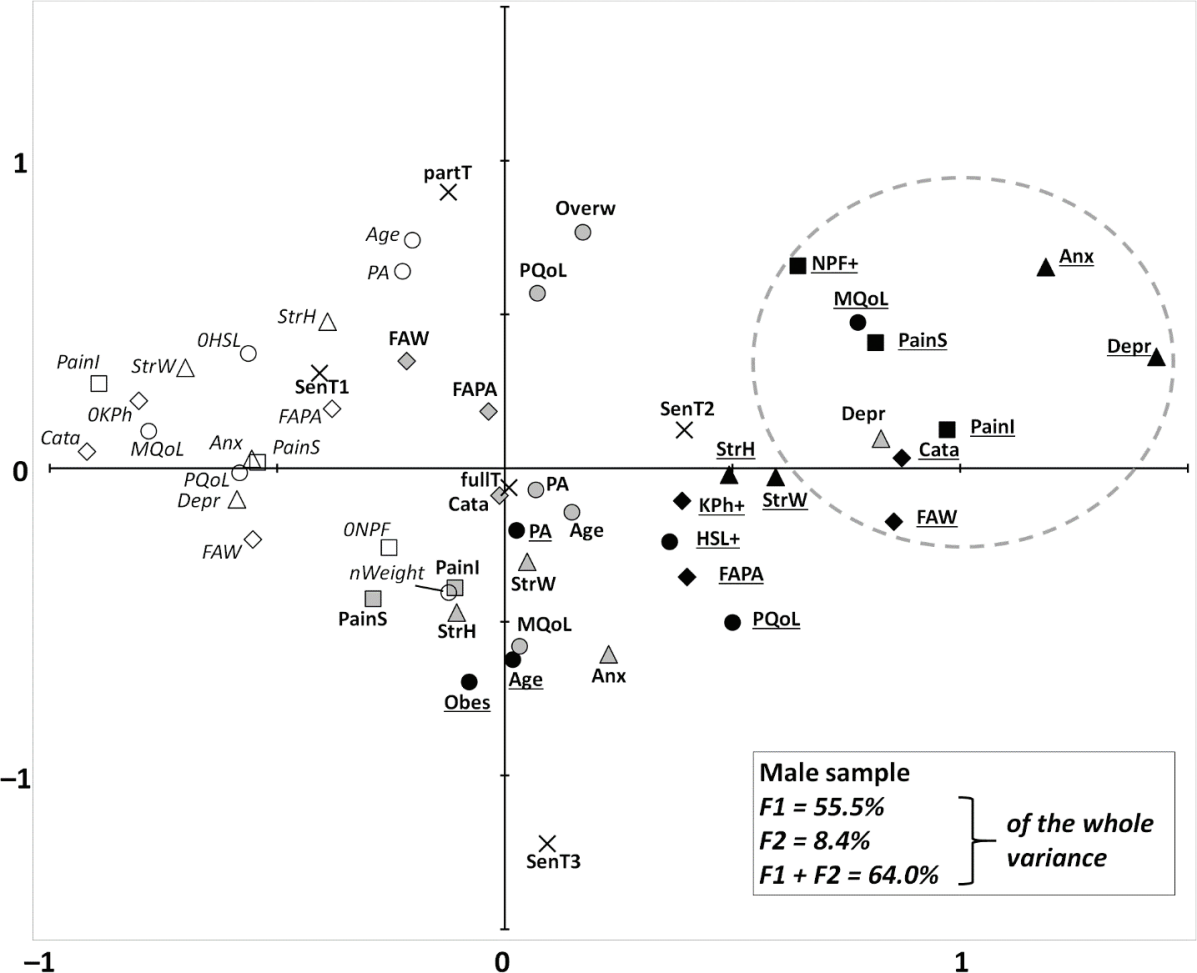
Multiple correspondence analysis of all the male cases who reported chronic low back pain (*n* = 92). The description is the same as for Fig. 1.

The MCA conducted on the male sample showed results with a similar pattern to the female sample, with respective contributions of F1 and F2 of 55.5% and 8.4% (64.0% for F1 + F2) (Fig. 2). However, the modalities of the highest disorders appeared more concentrated. The results of the cluster analyses are given in Table SIII and illustrated in Fig. 2. The final cluster analysis showed that high pain interference neighboured (by order of proximity): high pain catastrophizing, intermediate depression, high FAW, high pain severity, low mental QoL, high depression, high anxiety, and neuropathic features.

Finally, several significant relationships between work-related factors and pain interference were found, with noticeable sex differences. Not having consecutive rest days was associated with pain interference (*p* = 0.009), but this effect was significant only for women (*p* = 0.011). For women, pain interference was significantly associated with back torsion (*p* = 0.017), exposure to vibrations (*p* = 0.039), moving heavy loads (*p* = 0.0007), and a static standing position (*p* = 0.049); most of these gestures were related to a history of sick leave and to the perception of work as the cause of LBP or worsening it (data not shown). For men, none of these relationships were found to be significant.

## DISCUSSION

Our analyses show that in subjects surveyed at work and not consulting for this purpose, cLBP fits a biopsychosocial model, as illustrated by the interrelationships between high cLBP severity and interference, low QoL, affective and cognitive disorders, and some other work-related outcomes. In particular, modalities of high cLBP disorder neighboured high pain catastrophizing and neuropathic pain features for both sexes. However, the most interesting information is the different interrelationships in men and women; for men, high cLBP disorder was close to mental distress (high anxiety and depression, and low mental QoL), while for women it was close to low physical QoL, high FAPA and kinesiophobia, and high stress at work.

The strong relationship between high cLBP disorder and high pain catastrophizing was expected, but our cross-sectional design did not allow us to separate the role of baseline catastrophizing at the onset of cLBP, and that of the longitudinal interaction between cLBP and cLBP-aggravated catastrophizing. However, a recent sex- and age-matched cohort study of 71 cLBP patients showed that catastrophizing measured before a rehabilitation programme predicted improvement in pain intensity and disability, and that the decrease in catastrophizing during the programme was correlated to the above-mentioned improvements (7). This highlights the interest in assessing this aspect in cLBP patients.

The reported level of almost 30% of neuropathic features for the whole sample, and their association with high pain interference and severity, is of note. Although we could not provide clinical confirmation, or identify the respective contribution of lower limbs and/or of radiating back pain (22), this suggests that a non-negligible number of our cLBP cases had an underlying radicular component. Although disc degeneration and spinal stenosis are thought to be involved in radicular pain (1, 32), it is unknown whether our sample group of a middle-aged European population working in a French service business was particularly exposed to spinal lesions. Although carrying heavy burdens and frequent bending are risk factors in the workforce (31), such exposure has been largely reduced in Western countries thanks to mechanization; these preventative measures are currently applied in the company surveyed here, though occasional manual carrying is unavoidable. On the other hand, LBP is more frequent in high- than in middle/low-income countries, despite hard physical work being much less common (1), illustrating that low physical activity is also a risk factor for cLBP (29, 32–34).

The role of sex in the pathogenesis of cLBP is a complex topic. Much epidemiological data reports that more women are affected by LBP regardless of age (1), and this was confirmed for cLBP in a large Spanish cohort study (35). A recent review and another large Spanish cohort study also found the female sex to be a factor for chronicity in LBP (3, 34), and in another Spanish sex-matched study a higher lifetime prevalence of LBP was also found for women (36). This could explain the higher female rate of cLBP in our sample vs the non-painful responders. Anatomical, hormonal, neurobiological, and psychosocial factors are among the potential contributors to this predisposition to cLBP and kinesiophobia in women. Compared with men, cLBP women display more hypersensitivity to sensory testing (37, 38), and women complaining of pain on lumbar flexion have a lower QoL (39). More generally, in chronic musculoskeletal pain, central sensitization is more frequent in women than in men (40). Mechanical factors are also probably more commonly involved for women in the pathogenesis of cLBP. A meta-analysis of 18 studies conducted on nurses reported that, besides being a woman, being overweight was also a risk factor for cLBP (33). Another study conducted on 459 Serbian students showed that several self-perceived triggers for LBP, including poor body posture and lack of exercise, were more often reported by women (41). These studies suggest that biomechanical determinants of cLBP are more closely involved for women, and this is supported by our observation of the proximity of high cLBP disorder to low physical QoL in women, along with higher cognitive affects towards movement (i.e., kinesiophobia and FAPA). This was further supported by our analysis of the relationships with repetitive gestures that are harmful for the back and influence cLBP, which were found only in women, while those gestures were more often reported by men.

If we focus on the work environment, our observations can be compared with those of a French study, which followed 12,591 French workers in various occupations; of this cohort (36% women), 11.6% of the men and 6.2% of the women had developed LBP between 1990 and 1995 (14). The risk factors for developing LBP depended on sex, but the only relevant differences were for physical response to vibration (found only for women), while posture and strong physical effort were weaker and sex-independent factors. Also, the percentage of overweight cases was similar to our cLBP sample, although BMI did not influence LBP regardless of sex. Conversely, while men in our sample had higher BMIs than women, BMI was positively correlated with cLBP interference and intensity in women only. These discrepancies with the current study might be due to the 23-year gap between the 2 surveys, and the resultant change in occupations in between, with a tendency towards less physical effort, although with a greater requirement to stand to communicate with customers, or to bend over repeatedly to pick up objects. This suggests a role of anatomical and postural factors in this sex effect. Indeed, women – especially younger women – have a greater lumbar lordosis than men, which is particularly marked for standing occupations (42, 43). Furthermore, due to a gendered aim of sociability, women are more likely to adopt self-inflicted straightness when maintaining a standing posture (44).

A cohort study involving 607 Finnish metal industry employees showed that for men, but not for women, stressed and depressive states could predict LBP 10 years ahead (45). Analyses were adjusted for age and job (white- vs blue-collar workers) but not conducted within each of these 2 work categories. These results are consistent with ours for the men, and further supported by another study of 519 cLBP patients referred to a pain clinic in the USA, in which pain levels and psychological symptoms were more highly correlated in men than in women (46). However, none of these observations matches the common belief that there is a strong relationship between cLBP and occupational biomechanical factors in men. A possible explanation can be found in social changes in Western countries, where despite the persistence of sex segregation in many jobs, the sex ratio tends to be fairly balanced in the workforce, especially in the service sector (47). However, in a paradigm where, despite a persistent gendered perception of the role of work and work–life balance (48), men and women have similar jobs in general, sex might affect interrelationships within the biopsychosocial model. Such an effect could be particularly strong in the case of chronic pain: for example, working men affected by cLBP might feel more challenged, in terms of their social requirement to be a breadwinner, than women working in similar conditions, and therefore could be more mentally affected.

The current study has limitations, because we worked on a convenience sample size, as the cross-sectional design does not allow causality hypotheses to be drawn (e.g., whether psychological disorders were upstream or downstream, or both) and because our results cannot necessarily be extended to other work environments and countries. Also, although many parameters were well balanced between the men’s and the women’s sample groups (including age), men displayed a higher BMI and psychological distress, and reported repetitive gestures more often (including some involving the back). Finally, the participant’s declaration of work perceived as a cause of pain was only declarative and did not mean that work was actually a cause of pain, even partially.

To summarize, we observed that, taken at a relatively early stage of the disease (as they were all still at work, even though some of them had experienced sick leave), cLBP patients already followed the currently known biopsychosocial model. This encourages the use of biopsychosocial screening for workers at this early stage, before cLBP has evolved into a refractory state and before the patient’s relationship with work has reached a point of irreversible degradation. Either as a direct primer or through psychosocial mediators, work has a complex relationship with cLBP, which is supported by the proximity we observed, in our overall sample, between cLBP severity/interference, pain catastrophizing, and fear/avoidance towards work. A growing part of the research on cLBP lies nowadays in a cross-sectional definition of biopsychosocial profiles (phenotyping) (49, 50) and in longitudinal identification of evolutionary profiles (trajectories) depending on the phenotype (6, 51). The aim is to improve understanding of the mechanisms of treatment failure, and to build personalized programmes of care to improve efficacy (52). Large cohorts offer the possibility to build predictive models of efficacy (51), but our results suggest that such cohorts should be launched at the earliest possible stage of cLBP, i.e., before the first sick leave. Such early assessment could also help guide personalized care to prevent long-term cLBP and sick leave (3, 27). Nowadays, although exercise is still a primary intervention, an interdisciplinary approach has a growing place in rehabilitation programmes, in which self-care and pain neuroscience education empower patients by improving their understanding of the disease and their ability to manage symptoms and psychosocial factors (53). Such programmes can be integrated early in the care pathway and tailored to individual biopsychosocial profiles (and expected trajectories) (1, 5). For example, psychological follow-up or relaxation-based approaches could target patients presenting with predominantly psychological issues, whereas a more biomechanical and movement-oriented approach may be more appropriate in those with primarily physical complaints. Moreover, if work-related problems appear to be central, care should focus on ergonomic adjustments, targeted education on posture and movement, and psychosocial support, especially in sectors with repetitive physical tasks or high emotional demands. The reporting of neuropathic features should also be systematic for phenotyping, as already carried out by some personalized programmes (52). Finally, as we clearly identified a different biopsychosocial pattern based on sex, this must be integrated into our proposals for research and care. Although generalizations regarding sex-based differences should be made with caution, this indicates that phenotype-based predictive models should also be designed by sex group. For example, in cLBP patients, differences between sexes have already been reported for brain function (54), distress-mediated effect of analgesic drugs (55), or physical predictors of sick leave (27). Such an approach that takes sex into account is likely to improve the reliability of research results and the efficacy of care.

In conclusion, our findings bring an innovative concept to the broad field of mechanisms and treatment of cLBP, which still constitutes a major burden for the patients and for the society as a whole. They may help develop personalized prevention and intervention strategies in occupational settings, ultimately reducing the burden of cLBP. Future studies should investigate these sex-specific mechanisms in prospective cohorts and across diverse professional environments.

## Supplementary Material




