# Supplementary material for: MALE AND FEMALE WORKERS SUFFERING FROM CHRONIC LOW BACK PAIN DISPLAY DIFFERENT INTERRELATIONSHIPS BETWEEN THE BIOPSYCHOSOCIAL VARIABLES
Source: J Rehabil Med. 2025 Sep 4;57:43450. doi: 10.2340/jrm.v57.43450 (PMC12421334; doi:10.2340/jrm.v57.43450)
Supplement: Supplementary file 1 [file JRM-57-43450-s1.pdf]

Supplementary material has been published as submitted. It has not been copyedited, or typeset by Journal of Rehabilitation Medicine

Methods: supplementary information

A. Design and conduction of the survey

The survey aimed at targeting a sample representative of the whole workforce of IKEA France, which had 41 stores nationwide when the current study was designed. For this purpose, the board of IKEA France firstly selected 14 stores which characteristics were the same of the whole in terms of activity, size (building and staff), surrounding socioeconomic background, and date of creation. Secondly, as recruitment was based on the voluntary nature of the participants, the survey was divided into four waves of one month each, which were separated by 3-month breaks. An interim analysis of the distribution of jobs, age, and sex ratio was conducted at the end of each session. Each new wave was preceded by a communication campaign, in which the still underrepresented job types, if any, were specifically targeted.

The survey was launched after a preliminary communication campaign. Data collection was made using the REDCap electronic data capture tools, in accordance with French and European laws and regulations on data protection. The internet link allowing access to this platform was sent by the human resources department of each store for distribution to all employees. The only restrictive condition for participation was being able to read and understand French. Each participant wishing to participate was invited to log on to the dedicated REDCap electronic platform and to create an anonymous account; this could be done either at the workplace or at home. The participant then gained access to the information document detailing the purpose, content, and conduct of the study. Subsequently, the participant was asked to register consent, and was free to withdraw consent at any time by informing the sponsor.

As stated in the main publication (1), the demographic data of the survey sample (with or without chronic pain) and the wide ranging representation of different types of job were relatively close to those of the French working population (data from the latest INSEE [French institute of statistics and economic studies] report of 2016 (2).

B. *Content of the survey and questionnaires*

a. for all participants

Sociodemographic variables: sex, age (yrs), weight (kg), height (cm).

"Do you usually have physical activity?" (yes/no)

"If yes, please indicate the average number of hours a week" (1/2/3/4/5/>5)

"Which is your job in the company?" (sales / logistics / catering / fittings / administration / recovery / maintenance /cash register / home delivery / caddy gathering / other)

"Since how many months do you work in the company?"

"Since how many months do you work in this job?"

"How many hours do you work in a week (in average)?"

"Does your job schedule allows two consecutive rest days?" (yes/no)

"How many minutes do you take from home to your workplace?"

"Have you had any change of workstation since joining the company?" (yes/no)

"Does your job includes any of these repetitive gestures?" (yes/no; several possible "yes" responses) (arms/shoulders raised / wrist torsion / wrist flexion / neck torsion / neck flexion / back torsion / back flexion / exposure to vibrations / moving heavy loads / static standing position / static sitting position / extended work on screen)

The anxious and the depressive state were assessed by the Hospital Anxiety and Depression scale (HADS), which has been developed by Zigmond and Snaith in 1983 (3). The HADS is a 14-item scale, with 7 items relating to anxiety and 7 relating to depression. Each item on the questionnaire is scored from 0 to 3, so a person can score between 0 and 21 for either anxiety or depression. The HADS was used in its validated French version (4).

Pain catastrophizing is the tendency to describe pain experience in more exaggerated terms than the average person ("magnification"), to ruminate on it more ("rumination"), and/or to feel more helpless about the experience ("helplessness"). A higher pain catastrophizing predicts higher pain intensity levels for the same painful condition. It was assessed by the Pain Catastrophizing Scale (PCS), which has been developed by Sullivan et al. in 1995 (5). The PCS is a 13-item scale, and each item on the questionnaire is scored from 0 to 4, so a person can score between 0 and 52. Higher scores are associated with a higher pain catastrophizing. We did not use the subscales related to the three dimensions of catastrophizing. The PCS was used in its validated French version (6).

Psychological stress is a feeling of emotional strain and pressure, and is a form of mental discomfort. It results from perceptions either internal or external, i.e. related to the environment. We assessed the subject's perception of his/her own stress at work and at home by two consecutive questions, which were "At which level do you estimate your level of stress when (at work / at home), on the following scale? Please set the cursor at the best appropriate place." Such assessment had already been used previously (7,8). The subject had to place a cursor on an electronic visual analogue scale from "no stress at all" to "unbearable stress", and the position was then automatically converted into a scale from 0 to 100 and registered so.

b. for the participants who declared having pain lasting for more than 3 months

Chronic pain, in terms of intensity and of impact on day life ("interference"), was assessed by the Brief Pain Inventory (BPI), a questionnaire developed by the Pain Research Group of the WHO Collaborating Centre for Symptom Evaluation in Cancer Care in 1994 (9). Pain severity is addressed by 4 numerical rating scales to quote (i) the worst and the least pain felt in the last 24 hours, (ii) the average pain, and (iii) the pain currently felt ("right now"); a resulting pain severity score is calculated by averaging those 4 scores; we adjusted to a 0-to-10 range. Pain interference is addressed similarly by 7 numerical rating scales to quote the level of impairment on general activity, mood, walking ability, normal work (including housework), relations with other people, sleep, and enjoyment of life; a resulting pain interference score is calculated by averaging those 7 scores; we adjusted to a 0-to-10 range. For each of the 11 items, the numerical rating scales were converted into an electronic visual analogue scale as described above. In addition, the BPI offered a body map to help the patient at localizing his/her worst chronic pain. Of note, tools had been developed to assess specifically low back pain intensity and interference (disability), but we preferred the BPI in the current survey, as its original aim was to spot any kind of chronic pain. The BPI was used in its validated French version (10).

Whether or not chronic pain can be suspected as neuropathic can be estimated with the DN4 questionnaire, which is the best known screening tool for this purpose (11). The original version (in French) has 7 interview-based items and 3 examination-based items related to sensory and pain descriptors; among them, electric shocks or pins-and-needles sensations, numbness/hypoesthesia in the painful area, and whether light brushing increases or causes pain (allodynia), are the most typical signs observed in peripheral neuropathic pain. A positive response to 4 items is sufficient to define the DN4 as positive, i.e. possible neuropathic pain. In cLBP, the DN4 has good screening properties for neuropathic pain either restricted to the back or radiating pain in the lower limb; the rate of positivity is almost null in patients with pain restricted to the lumbar area, 15% in patients with pain radiating proximally, 39% in patients with pain radiating below the knee without neurologic signs, and 80% in patients with pain radiating towards the foot in a dermatomal distribution, with neurological

signs, corresponding to typical radiculopathy (12). For the current survey, the DN4 was here fitted to patient interview, which means that only the 7 interview-based items were offered, with a cut-off value lowered to 3 (11,13).

Health-related quality-of-life is how a patient's well-being may be affected over time by a disease, disability or disorder; we assessed it with the 12-item Short Form survey (SF-12) of the Medical Outcomes Study, which has been developed by Gandek et al. in 1998 (14). The SF-12 has 12 items and covers 8 domains of quality-of-life: limitations in physical activities because of health problems, limitations in social activities because of physical or emotional problems, limitations in usual role activities because of physical health problems, bodily pain, general mental health, limitations in usual role activities because of emotional problems, vitality, and general health perceptions. An algorithm builds two summary measures, the Physical Component Summary (PCS) and the Mental Component Summary (MCS), both ranging from 0 to 100 (worst to best). The SF-12 was used in its validated French version (14).

Kinesiophobia is the fear of physical movement and activity resulting from a feeling of vulnerability to painful injury or reinjury; it was assessed by the Tampa Scale of Kinesiophobia (TSK), developed in 1991 (15), then improved for psychometrical purposes (16). The TSK is a 17-item scale, and the items are scored from 1 to 4 (or 4 to 1 for four negatively worded items), so a person can score between 17 (no/negligible kinesiophobia) and 68, the higher scores indicating a higher kinesiophobia. The TSK was used in its validated French version (17); a score over 40 signals a relevant kinesiophobia.

The Fear-avoidance Beliefs Questionnaire (FABQ) has been developed in 1993 by Waddell et al. (18), to assess the patients' beliefs about how physical activity and work affected their low back pain. The FABQ consists in 16 questions, of which 5 are not used to calculate scores; each item is answered on a 7-point Likert scale (0= completely disagree, 6=completely agree). It explores two dimensions, fear/avoidance towards physical activity ("FAPA", 4 items, score ranging from 0 to 24), and fear/avoidance towards work ("FAW", 7 items, score ranging from 0 to 42); a higher score indicates a higher fear/avoidance. Cut-off values for FAPA and FAW had been reported, but from a study recruiting only the most severe cases of cLBP (19). The FABQ was used in its validated French version (20).

Additional questions were offered to the participant to self-define various aspects of his/her pain:

- "Which factors do you think they are the cause of your pain (e.g. sport, gesture at work, traumatism, surgery…)? (free text field)
- "Do you think your pain is related to your job?" (yes/no, + free text field for more details)

- “Has you pain already led to a sick leave?” (yes/no)
- “Has you pain already led to a layout or change of workstation?” (yes/no)
- “Do you think your pain impairs your quality of life at work?” (yes/no)

Along with the responses to the items of the DN4 and to the body map of the BPI, the responses to all these questions were considered by the pain physician to define the case as LBP and the features of pain (nociceptive, neuropathic, nociplastic, or mixed).

C. Univariate analyses

Those analyses were conducted prior to multiple correspondence analyses (MCAs) to identify the trends in interrelationships between the biopsychosocial variables and the collinearities to discard the redundant variables. Those analyses – in which each variable was tested against each other – were conducted within the whole sample and within each sex group. Inferences were carried out only to highlight the strongest associations, and the type-I error inflation was not corrected. The analyses were harmonised in order to build correlation matrixes able to show all the associations in one framework. For such harmonisation, nominal variables were transformed into numerical ordinal ones; binary variables (yes/no) became 1/0. Also, in order to show positive rather than negative correlations with cLBP outcomes, some variables were converted to their opposite; to do so, both QoL summary components (mental and physical) were converted into parameters of 'impaired QoL' by subtracting the score from 100 (the maximal possible value) for each subject, and the ordinal modalities of the variable "physical activity" were inversed to create a "low physical activity" variable. As none of the numerical variables followed a Gaussian distribution (apart from BMI), the univariate analyses were all Spearman's correlations, expressed as the ρ coefficient whose difference from the null value was statistically tested. Each univariate correlation analysis was interpreted by reading from side-to-side both the effect size (expressed by the ρ coefficient) and the statistical significance.

D. Cluster analyses

These analyses studied the proximity between the different modalities of the different variables, which position was defined by their respective principal coordinates on the main factorial axes of the MCA (F1 and F2). The final aim was to identify the modalities representing the states of highest disorder/risk, which are those neighbouring the modality of highest pain interference (3rd tercile).

Three clustering methods were used in parallel:

- the ascending hierarchical classification, in which dissimilarities were defined by the Euclidian distances between points (modalities), and aggregation was made by Ward's linkage method, with no *pre hoc* definition of the number of cuts on the dendrogram;
- the k-means method, in which the number *k* of clusters was set at 3 according to the expected partition into 3 levels of disorder/risk;
- the univariate clustering was made on the Euclidian distances between the modality of highest pain interference (3rd tercile) and each other modality of the diagram; the number of clusters was also set at 3.

The final cluster representing the worst state of cLBP included those modalities which belonged to the cluster including the highest pain interference (3rd tercile) in the three methods.

E. References

(1) Kerckhove N, Lambert C, Corteval A, Pereira B, Eschalier A, Dualé C. Cross-sectional study of prevalence, characterization and impact of chronic pain disorders in workers. J Pain 2021 ; 22 : 520-532. https://doi.org/10.1016/j.jpain.2020.11.005.

(2) INSEE. Tableaux de l'Économie Française – Édition 2016 – Population active. https://www.insee.fr/fr/statistiques/1906671?sommaire=1906743#tableau-T16F041G2 . [accessed 20-8-2019].

(3) Zigmond AS, Snaith RP. The hospital anxiety and depression scale. Acta Psychiatr Scand 1983 ; 67 : 361-370. https://doi.org/10.1111/j.1600-0447.1983.tb09716.x.

(4) Lépine JP, Godchau M, Brun P, Lempirière P. Évaluation de l'anxiété et de la dépression chez des patients hospitalisés dans un service de médecine interne. Ann Med Psychol (Paris) 1985 ; 2 : 175-185. https://doi.org/https://psycnet.apa.org/record/1988-70244-001.

(5) Sullivan MJ, Bishop S, Pivik J. The pain catastrophizing scale: development and validation. Psychol Assessment 1995 ; 7 : 524-532. https://doi.org/10.1037//1040-3590.7.4.524.

(6) French DJ, Noël M, Vigneau F, French JA, Cyr CP, Evans RT. L'échelle de dramatisation face à la douleur PCS-CF. Adaptation canadienne en langue française de l'échelle «Pain Catastrophizing Scale». Can J Behaviour Science 2005 ; 37 : 181-192. https://doi.org/10.1037/h0087255.

(7) Lesage FX, Berjot S. Validity of occupational stress assessment using a visual analogue scale. Occup Med (Lond) 2011 ; 61 : 434-436. https://doi.org/10.1093/occmed/kqr037.

(8) Lesage FX, Berjot S, Deschamps F. Clinical stress assessment using a visual analogue scale. Occup Med (Lond) 2012 ; 62 : 600-605. https://doi.org/10.1093/occmed/kqs140.

(9) Cleeland CS, Ryan KM. Pain assessment: global use of the Brief Pain Inventory. Ann Acad Med Singap 1994 ; 23 : 129-138

(10) Poundja J, Fikretoglu D, Guay S, Brunet A. Validation of the French version of the brief pain inventory in Canadian veterans suffering from traumatic stress. J Pain Symptom Manage 2007 ; 33 : 720-726. https://doi.org/10.1016/j.jpainsymman.2006.09.031.

(11) Bouhassira D, Attal N, Alchaar H, Boureau F, Brochet B, Bruxelle J et al. Comparison of pain syndromes associated with nervous or somatic lesions and development of a new neuropathic pain diagnostic questionnaire (DN4). Pain 2005 ; 114 : 29-36. https://doi.org/10.1016/j.pain.2004.12.010.

(12) Attal N, Perrot S, Fermanian J, Bouhassira D. The neuropathic components of chronic low back pain: a prospective multicenter study using the DN4 Questionnaire. J Pain 2011 ; 12 : 1080-1087. https://doi.org/10.1016/j.jpain.2011.05.006.

(13) Ragusa C, Pereira B, Balayssac D. Study of the relationship between psychoactive substance use and pain in cancer patients and cancer survivors: A French nationwide cross-sectional study. Int J Cancer 2024 ; 155 : 1078-1090. https://doi.org/10.1002/ijc.35006.

(14) Gandek B, Ware JE, Aaronson NK, Apolone G, Bjorner JB, Brazier JE et al. Cross-validation of item selection and scoring for the SF-12 Health Survey in nine countries: results from the IQOLA Project. International Quality of Life Assessment. J Clin Epidemiol 1998 ; 51 : 1171-1178. https://doi.org/10.1016/s0895-4356(98)00109-7.

(15) Miller RP, Kori SH, Todd DD. The Tampa scale - a measure of kinesiophobia. Clin J Pain 7, 51. 1991.

(16) French DJ, France CR, Vigneau F, French JA, Evans RT. Fear of movement/(re)injury in chronic pain: a psychometric assessment of the original English version of the Tampa scale for kinesiophobia (TSK). Pain 2007 ; 127 : 42-51. https://doi.org/10.1016/j.pain.2006.07.016.

(17) French DR, Mayes PJ, Mayes S. Peur du mouvement chez des accidentés du travail: l'Échelle de Kinésiophobie de Tampa (EKT). Can J Behav Sci 2002 ; 34 : 28-33. https://doi.org/https://doi.org/10.1037/h0087152.

(18) Waddell G, Newton M, Henderson I, Somerville D, Main CJ. A Fear-Avoidance Beliefs Questionnaire (FABQ) and the role of fear-avoidance beliefs in chronic low back pain and disability. Pain 1993 ; 52 : 157-168. https://doi.org/10.1016/0304-3959(93)90127-B.

(19) George SZ, Fritz JM, Childs JD. Investigation of elevated fear-avoidance beliefs for patients with low back pain: a secondary analysis involving patients enrolled in physical therapy clinical trials. J Orthop Sports Phys Ther 2008 ; 38 : 50-58. https://doi.org/10.2519/jospt.2008.2647.

(20) Chaory K, Fayad F, Rannou F, Lefevre-Colau MM, Fermanian J, Revel M et al. Validation of the French version of the fear avoidance belief questionnaire. Spine (Phila Pa 1976) 2004 ; 29 : 908-913. https://doi.org/10.1097/00007632-200404150-00018.
