# Supplementary material for: MALE AND FEMALE WORKERS SUFFERING FROM CHRONIC LOW BACK PAIN DISPLAY DIFFERENT INTERRELATIONSHIPS BETWEEN THE BIOPSYCHOSOCIAL VARIABLES
Source: J Rehabil Med. 2025 Sep 4;57:43450. doi: 10.2340/jrm.v57.43450 (PMC12421334; doi:10.2340/jrm.v57.43450)
Supplement: Supplementary file 2 [file JRM-57-43450-s2.pdf]

Supplementary material has been published as submitted. It has not been copyedited, or typeset by Journal of Rehabilitation Medicine

Table SI. Spearman correlation matrix (whole sample, N=256; female and male patients separately, n=164 and 92, respectively).

	Sex	Age	Seniority	Time spent at work	Low physical activity	BMI	History of sick leave	Impaired physical QoL	Pain severity	Neuropathic features	Pain interference	Kinesiophobia	FAPA	FAW	Catastrophism	Depression	Anxiety	Impaired mental QoL	Stress at work	Stress at home
**Age**			**0.491**	0.006	0.116	0.025	**0.187**	**0.152**	-0.003	0.044	0.042	0.005	-0.003	-0.054	-0.004	0.074	0.035	-0.065	0.051	-0.076
**Seniority**	**M**	**0.490**		-0.022	**0.165**	0.048	**0.147**	0.107	0.016	0.089	0.039	0.068	0.085	0.013	0.082	0.099	0.061	0.054	**0.157**	-0.031
	**F**	**0.496**																		
**Time (work)**	**M**	0.069	-0.076		-0.009	-0.089	-0.026	**-0.168**	-0.063	-0.032	-0.054	-0.035	0.069	0.027	0.020	-0.015	0.001	-0.011	0.066	-0.003
	**F**	-0.021	-0.005																	
**Low phys. activity**	**M**	0.114	**0.222**	-0.065		**0.127**	0.069	0.053	-0.003	0.017	0.044	0.024	0.080	-0.064	0.052	**0.189**	0.069	0.122	0.058	0.086
	**F**	-0.120	-0.136	0.004																
**BMI**	**M**	-0.066	0.171	-0.044	0.036		0.112	**0.148**	**0.244**	-0.008	**0.194**	0.094	0.003	0.121	0.038	**0.180**	-0.050	0.047	0.056	-0.061
	**F**	0.083	0.000	-0.142	**0.197**															
**Hist. sick leave**	**M**	0.114	**0.207**	-0.185	0.051	-0.062		**0.439**	**0.281**	0.079	**0.390**	**0.290**	0.097	**0.297**	**0.289**	**0.229**	**0.124**	0.093	**0.166**	0.030
	**F**	**0.222**	0.111	0.048	0.090	**0.214**														
**Impaired PhQoL**	**M**	**0.235**	0.190	**-0.274**	0.131	-0.072	**0.445**		**0.407**	**0.199**	**0.511**	**0.521**	**0.408**	**0.389**	**0.410**	**0.189**	0.082	**-0.206**	**0.224**	-0.106
	**F**	0.109	0.062	-0.127	0.033	**0.242**	**0.439**													
**Pain sever.**	**M**	0.037	0.005	-0.003	-0.157	0.083	0.188	**0.243**		**0.202**	**0.746**	**0.300**	**0.128**	**0.349**	**0.362**	**0.319**	**0.272**	**0.207**	**0.292**	**0.153**
	**F**	-0.013	0.024	-0.095	0.089	**0.306**	**0.337**	**0.487**												
**NPath feat.**	**M**	-0.109	-0.079	-0.041	0.052	0.095	-0.140	0.057	0.181		**0.185**	**0.268**	**0.183**	**0.281**	**0.193**	**0.162**	**0.238**	0.106	**0.211**	0.046
	**F**	0.119	**0.190**	-0.024	-0.006	-0.051	**0.200**	**0.273**	**0.214**											
**Pain interf.**	**M**	0.139	0.133	-0.069	-0.118	0.041	**0.404**	**0.445**	**0.646**	0.094		**0.460**	**0.299**	**0.408**	**0.555**	**0.493**	**0.423**	**0.313**	**0.344**	**0.157**
	**F**	-0.008	-0.015	-0.056	**0.154**	**0.252**	**0.386**	**0.541**	**0.793**	**0.226**										

**Kinesio-phobia**	**M**	-0.017	0.053	-0.154	0.071	0.065	**0.313**	**0.505**	**0.233**	**0.211**	**0.429**		**0.541**	**0.507**	**0.583**	**0.362**	**0.239**	**0.180**	**0.251**	-0.049
	**F**	0.010	0.076	-0.020	0.067	0.045	**0.283**	**0.543**	**0.321**	**0.320**	**0.476**									
**FAPA**	**M**	0.080	0.106	-0.003	**0.208**	-0.042	0.042	**0.320**	-0.067	0.122	**0.233**	**0.560**		**0.432**	**0.430**	**0.264**	**0.171**	0.102	**0.224**	-0.017
	**F**	-0.049	0.064	0.098	0.020	0.006	0.121	**0.450**	**0.210**	**0.227**	**0.332**	**0.520**								
**FAW**	**M**	-0.073	-0.034	0.072	0.085	0.014	**0.282**	**0.299**	**0.318**	**0.265**	**0.361**	**0.508**	**0.231**		**0.389**	**0.320**	**0.142**	**0.219**	**0.323**	-0.067
	**F**	-0.040	0.029	-0.032	-0.077	0.117	**0.315**	**0.436**	**0.383**	**0.309**	**0.436**	**0.428**	**0.492**							
**Catastro-phism**	**M**	0.075	**0.242**	-0.123	0.023	-0.036	**0.260**	**0.371**	**0.346**	**0.273**	**0.544**	**0.560**	**0.368**	**0.408**		**0.484**	**0.422**	**0.373**	**0.304**	0.099
	**F**	-0.044	-0.009	0.069	0.083	0.065	**0.304**	**0.427**	**0.369**	**0.159**	**0.561**	**0.602**	**0.450**	**0.370**						
**Depression**	**M**	0.062	**0.225**	-0.092	0.077	0.153	**0.295**	**0.394**	**0.423**	**0.361**	**0.582**	**0.430**	**0.297**	**0.388**	**0.609**		**0.599**	**0.651**	**0.354**	**0.254**
	**F**	0.082	0.038	-0.005	**0.296**	0.149	**0.200**	0.087	**0.267**	0.077	**0.437**	**0.285**	**0.234**	**0.250**	**0.427**					
**Anxiety**	**M**	0.093	0.106	-0.139	-0.055	0.114	**0.220**	0.145	**0.406**	**0.387**	**0.485**	**0.303**	0.187	0.122	**0.460**	**0.669**		**0.568**	**0.355**	**0.337**
	**F**	0.000	0.043	0.072	0.133	-0.103	0.072	0.046	**0.214**	0.139	**0.389**	**0.251**	**0.182**	**0.189**	**0.410**	**0.597**				
**Impaired MQoL**	**M**	-0.024	0.147	-0.054	0.064	0.121	0.151	-0.032	**0.345**	**0.303**	**0.437**	0.190	0.176	**0.337**	**0.521**	**0.709**	**0.636**		**0.370**	**0.346**
	**F**	-0.086	0.007	0.002	**0.170**	0.014	0.064	**-0.296**	0.136	-0.003	**0.241**	**0.162**	0.053	**0.160**	**0.292**	**0.628**	**0.536**			
**Stress at work**	**M**	**0.315**	**0.249**	0.062	0.129	0.008	0.102	**0.321**	**0.241**	**0.236**	**0.325**	**0.263**	**0.211**	**0.241**	**0.470**	**0.492**	**0.373**	**0.403**		**0.249**
	**F**	-0.081	0.102	0.042	0.049	0.046	**0.203**	**0.176**	**0.328**	**0.192**	**0.350**	**0.189**	**0.194**	**0.337**	**0.205**	**0.252**	**0.349**	**0.341**		
**Stress At home**	**M**	0.153	0.143	0.074	0.005	-0.014	0.121	0.023	**0.269**	0.076	**0.277**	0.092	0.028	0.093	**0.279**	**0.320**	**0.351**	**0.299**	**0.346**	
	**F**	**-0.185**	-0.132	-0.033	0.122	-0.070	-0.017	**-0.172**	0.099	0.025	0.090	-0.093	-0.030	-0.129	0.015	**0.234**	**0.322**	**0.371**	**0.205**	

In each case the ρ coefficient is shown for the correlation tested between the variable in the row header and that in the column header. Above and to the right of the diagonal are the coefficients for the whole sample, and below and to the left are the coefficients for female (F) and male (M) patients, separately. Significant differences from the null value are shown in bold. The abbreviations used for the row headers are explained by the column headers, presented in the same order. BMI, body mass index; FAPA: fear/avoidance towards physical activity; FAW: fear/avoidance towards work; QoL: quality of life.

Table SII. Clustering analyses (women's group, N=164)

Modality	Distance	Univariate	AHC	k-means
PainI (high) *	0.000	X	X	X
Cata (high) *	0.228	X	X	X
FAW (high) *	0.252	X	X	X
StrW (high) *	0.272	X	X	X
PainS (high) *	0.302	X	X	X
FAPA (high) *	0.341	X	X	X
KPh+ *	0.387	X	X	X
PhQoL (low) *	0.560	X	X	X
NPF+ *	0.572	X	X	X
Depr (intermediate) *	0.626	X	X	X
HSL+	0.635	X		X
Overw	0.785			
Depr (high)	0.799		X	X
Age (intermediate)	0.800			
Sen T2	0.801			
PA (low)	0.839			
Obes	0.886			
Anx (high)	0.886		X	X
MQoL (intermediate)	0.932			
PartT	0.945			
PainS (intermediate)	0.959			
Anx (intermediate)	0.970			
MQoL (low)	1.023		X	X
fullT	1.043			
Sen T3	1.052			
PhQoL (intermediate)	1.070			
StrW (intermediate)	1.076			
Cata (intermediate)	1.088			
PainI (intermediate)	1.106			
FAPA (intermediate)	1.133			
Age (high)	1.153			
StrH (low)	1.185		X	
nWeigh	1.190			
Age (low)	1.200			
StrH (low)	1.230			
StrH (intermediate)	1.233			
FAW (intermediate)	1.237			
NPF0	1.276			
PA (intermediate)	1.288			
Sen T1	1.307			
Depr (low)	1.338			
PA (high)	1.565			
KPh0	1.576			
Anx (low)	1.583			
FAPA (low)	1.657			

StrW (low)	1.658			
HSL0	1.677			
FAW (low)	1.758			
MQoL (high)	1.795			
PainS (low)	1.816			
Cata (low)	1.867			
PhQoL (high)	1.896			
PainI (low)	1.971			

Cluster analysis of the female surveyed cases who reported chronic low back pain (cLBP). In those clustering analyses, each variable was categorized into classes, and each of the resulting modalities was analysed through its principal coordinates on the main factorial axes of the Multiple Correspondence Analysis (MCA). The health-related (cLPB or else), affective and cognitive variables had been categorized into three classes, each corresponding to a level of disorder/risk (low, intermediate or high). The three classes of body mass index (BMI) are normal weight ('nWeigh'), overweight ('Overw') and obesity ('Obes'), with the same respective grade of risk. As neuropathic features and kinesiophobia were classified as presence ('+') or absence ('0'), they had no intermediate modality. The ascending hierarchical classification (AHC) and the k-means clustering methods analysed directly the coordinates, while the univariate clustering analysed the Euclidian distance between each modality and the modality of highest (3rd tercile) pain interference ('PainI (high)'). The symbol 'X' signals that the modality belongs to the same cluster as the 'PainI (high)' modality. The modalities are classified by decreasing order of Euclidian distance from the 'PainI (high)' modality. At the end, we considered as a final cluster representing relevantly the states of highest disorder/risk, those modalities which belonged to the same cluster as the 'PainI (high)' modality according to the three clustering methods; this final cluster is signalled by the symbol '*'. The variables directly related to chronic low back pain (cLBP) are: pain severity ('PainS') and pain interference ('PainI'), both from the Brief Pain Inventory; and neuropathic features ('NPF'). The affective variables are: anxiety ('Anx') and depression ('Depr') from the Hospital Anxiety and Depression scale; and stress at home ('StressH') and at work ('StressW'), both from analogue visual scales. The cognitive variables are: pain catastrophizing ('Cata') out of the Pain Catastrophizing Scale; fear/avoidance towards physical activity ('FAPA') and towards work ('FAW') from the Fear-Avoidance Back Questionnaire; and kinesiophobia from the Tampa Scale of Kinesiophobia. The other health-related variables are: the age, the declared physical activity ('PA'), the BMI, and the physical and mental quality-of-life (respectively 'PQoL' and 'MQoL'), which are the component summaries from the SF-12. The other work-related variables are: seniority within the job ('Sen', in terciles T1-2-3), and weekly time spent at work ("fullT" and "partT" respectively for full and partial time).

Table SIII. Clustering analyses (men's group, N=92)

Modality	Distance	Univariate	AHC	k-means
PainI (high) *	0.000	X	X	X
Cata (high) *	0.136	X	X	X
Depr (intermediate) *	0.149	X	X	X
FAW (high) *	0.321	X	X	X
PainS (high) *	0.324	X	X	X
MQoL (low) *	0.401	X	X	X
StrW (high)	0.407	X		X
StrH (high)	0.501	X		X
Depr (high) *	0.517	X	X	X
Anx (high) *	0.572	X	X	X
Sen T2	0.577	X		X
NPF+ *	0.625	X	X	X
KPh+	0.627	X		
HSL+	0.710	X		
FAPA (high)	0.745	X		
PhQoL (low)	0.784	X		
Age (intermediate)	0.867			
PA (intermediate)	0.924			
fullT	0.981			
PA (low)	1.002			
PhQoL (intermediate)	1.004			
Cata (intermediate)	1.007			
FAPA (intermediate)	1.009			
StrW (intermediate)	1.018			
Overw	1.027			
Anx (intermediate)	1.042			
MQoL (intermediate)	1.174			
PainI (intermediate)	1.197			
FAW (intermediate)	1.208			
Age (high)	1.212			
nWeigh	1.217			
StrH (intermediate)	1.231			
NPF0	1.284			
PA (high)	1.304			
Age (low)	1.327			
Obes	1.333			
partT	1.342			
FAPA (low)	1.353			
PainS (intermediate)	1.376			
Sen T1	1.392			
StrH (low)	1.407			
S PainS (low)	1.518			
Anx (low)	1.531			
HSL0	1.555			

PhQoL (high)	1.561			
FAW (low)	1.566			
Depr (low)	1.577			
Sen T3	1.607			
StrW (low)	1.686			
MQoL (high)	1.755			
KPh0	1.779			
PainI (low)	1.870			
Cata (low)	1.891			

Cluster analysis of the male surveyed cases who reported chronic low back pain (cLBP). In those clustering analyses, each variable was categorized into classes, and each of the resulting modalities was analysed through its principal coordinates on the main factorial axes of the Multiple Correspondence Analysis (MCA). The health-related (cLPB or else), affective and cognitive variables had been categorized into three classes, each corresponding to a level of disorder/risk (low, intermediate or high). The three classes of body mass index (BMI) are normal weight ('nWeigh'), overweight ('Overw') and obesity ('Obes'), with the same respective grade of risk. As neuropathic features and kinesiophobia were classified as presence ('+') or absence ('0'), they had no intermediate modality. The ascending hierarchical classification (AHC) and the k-means clustering methods analysed directly the coordinates, while the univariate clustering analysed the Euclidian distance between each modality and the modality of highest (3rd tercile) pain interference ('PainI (high)'). The symbol 'X' signals that the modality belongs to the same cluster as the 'PainI (high)' modality. The modalities are classified by decreasing order of Euclidian distance from the 'PainI (high)' modality. At the end, we considered as a final cluster representing relevantly the states of highest disorder/risk, those modalities which belonged to the same cluster as the 'PainI (high)' modality according to the three clustering methods; this final cluster is signalled by the symbol '*'. The variables directly related to chronic low back pain (cLBP) are: pain severity ('PainS') and pain interference ('PainI'), both from the Brief Pain Inventory; and neuropathic features ('NPF'). The affective variables are: anxiety ('Anx') and depression ('Depr') from the Hospital Anxiety and Depression scale; and stress at home ('StressH') and at work ('StressW'), both from analogue visual scales. The cognitive variables are: pain catastrophizing ('Cata') out of the Pain Catastrophizing Scale; fear/avoidance towards physical activity ('FAPA') and towards work ('FAW') from the Fear-Avoidance Back Questionnaire; and kinesiophobia from the Tampa Scale of Kinesiophobia. The other health-related variables are: the age, the declared physical activity ('PA'), the BMI, and the physical and mental quality-of-life (respectively 'PQoL' and 'MQoL'), which are the component summaries from the SF-12. The other work-related variables are: seniority within the job ('Sen', in terciles T1-2-3), and weekly time spent at work ("fullT" and "partT" respectively for full and partial time).
